# Relationship between resilience and death anxiety of the older adults during the coronavirus disease 2019 (COVID-19) pandemic

**DOI:** 10.1186/s12877-023-04086-8

**Published:** 2023-06-15

**Authors:** Fatemeh Rayatpisheh, Camellia Torabizadeh, Majid Najafi Kalyani, Zahra Farsi

**Affiliations:** 1 Student Research Committee, School of Nursing and Midwifery, Shiraz University of Medical Sciences, Shiraz, Iran; 2 Community Based Psychiatric Care Research Center, Department of Medical Surgical Nursing, School of Nursing and Midwifery, Shiraz University of Medical Sciences, Shiraz, Iran; 3 Community Based Psychiatric Care Research Center, Department of Medical Emergencies, School of Nursing and Midwifery, Shiraz University of Medical Sciences, Shiraz, Iran; 4 Research and Community Health Departments, Nursing School, Aja University of Medical Sciences, Tehran, Iran

**Keywords:** Resilience, Death anxiety, Older adult, Coronavirus disease 2019

## Abstract

**Background:**

The outbreak of coronavirus disease 2019 (COVID-19) caused an increase in the incidence of physical and psychological problems, particularly in the older adult. Considering the special physical and mental health conditions of the older adult, they were more exposed to psychological problems associated with the pandemic, such as death anxiety. Therefore, assessing the psychological status of this group is necessary in order to implement appropriate interventions. The present study aimed to investigate the relationship between resilience and death anxiety in the older adult during the COVID-19 pandemic.

**Methods:**

This descriptive-analytical study was conducted on 283 older adult people over the age of 60 years. The older adult population was selected from 11 municipal districts of Shiraz, Iran, using the cluster sampling method. The resilience and death anxiety scales were used for data collection. Data analysis was performed in SPSS version 22, using Chi-square test, t-test, and Pearson’s correlation coefficient test. A P-value less than 0.05 was considered statistically significant.

**Results:**

The mean and standard deviation of the older adult’s resilience and death anxiety scores were 64.16 ± 9.59 and 6.3 ± 2.95, respectively. There was a significant correlation between resilience and death anxiety scores (P < 0.01, r=-0.290). Also, sex (P = 0.00) and employment status (P = 0.00) were significantly associated with the older adult’s resilience. Besides, sex (P = 0.010) and employment status (P = 0.004) were significantly related to death anxiety.

**Conclusions:**

Our findings showcase levels of resilience and death anxiety in older adults during the covid-19 pandemic and suggest that resilience and death anxiety are inversely linked. This has implications on policy planning for future major health events.

## Background

The coronavirus disease 2019 (COVID-19) pandemic has caused an increase in the prevalence of mental health problems, such as anxiety, depression, antisocial behaviors, and irritability around the world [1–4]. The psychological problems of individuals during the COVID-19 pandemic were mainly caused by the pandemic itself rather than COVID-19-related deaths [5]. The older adult population, as a vulnerable group, was exposed to serious harms and risks, including psychological problems, because of adverse physical and psychological conditions during this period [6, 7].

Death anxiety, as one of the common psychological problems, is defined as fear and anxiety caused by thoughts of one’s own death or relatives’ death and is characterized by negative feelings, such as distress, anxiety, worry, and fear of death [8]. Persistence and severity of death anxiety can cause adverse health outcomes, such as physical problems, low resilience, and other mental health issues, including depression, generalized anxiety, phobia, panic, and suicidal ideation [9–12]. Based on terror management theory (TMT) awareness of death caused fear and anxiety and individuals use of a wide ranges of defense mechanisms against it [13].

The COVID-19 pandemic caused fear and anxiety in the older adults [14] and exposed them to death anxiety more than other age groups over a long period [15]. Owing to a decline in the immune system function of the older adult, they show poor immune responses to diseases and infections. Besides, since underlying diseases are more prevalent in the older adult compared to other age groups, their susceptibility to COVID-19 infection and death is higher [16]. The risk of infection and death due to COVID-19 is three times higher in the older adult than the youth [17]. Because of the high transmissibility rate of this virus, home quarantine is the first and most effective strategy to control its transmission [18].

Considering the special physical conditions of the older adult compared to other age groups, they were more susceptible to COVID-19; consequently, home quarantine and social isolation were even stricter in this group [19]. During the pandemic, home quarantine and reduced social interactions led to reduced physical activities and social relations in the older adult and threatened their mental health [20]. All the mentioned factors caused a significant increase in the prevalence of psychological disorders, such as death anxiety, among the older adult during the COVID-19 pandemic compared to normal conditions [21]. Thus, Older adults are vulnerable and must be protected [22].

Resilience is adaptability or psychological adaptation. It can be defined as a dynamic process, capacities, and outcomes of individual development in specific populations, situations, and risks [23]. Resilience can be effective in promoting mental health and managing psychological problems in adverse conditions [24]. High resilience is associated with a decline in psychological problems, such as depression, anxiety, and obsession [25]. Overall, resilience is a compatible construct that enables individuals to show flexibility in times of adversity [26]. An individual with adequate resilience does not suppress his/her feelings, but manages them in the best way possible under stressful conditions and actively attempts to find an effective solution [27]. Evidence suggests that resilience is linked to death anxiety and life expectancy [28], optimism and distress [29], anxiety [30, 31], worry about contracting COVID-19 [32], and depression [33]. Also, it has been found that tolerance based teaching to the older adult reduce their anxiety and increase their life expectancy [34]. Previous studies showed that the relation of resilience with health behavior was mediated by problem –focused coping [35] and stress in relation with risk of contracting COVID-19 [36].

Since the older adult people constitute a large proportion of the world’s total population, and death anxiety is one of the critical challenges of this population during the COVID-19 pandemic, it is essential to conduct relevant studies and identify influential factors in the management of mental disorders in these individuals. On the other hand, considering the limited number of relevant studies according to our literature review, the current research aimed to investigate the relationship between resilience and death anxiety in the older adult during the COVID-19 pandemic.

## Methods

### Design

This descriptive-analytical study was conducted between July and September 2022. The study setting included the health centers of Shiraz (Iran). Seven out of 11 municipal districts of Shiraz were selected via cluster sampling; the study population consisted of all the older adult presenting to these health centers.

### Sample size

Given the absence of similar research during the COVID-19 pandemic, a sample size of 258 people was measured with an effect size of 0.2. Considering an attrition rate of 10%, the sample size was increased, and the final sample size was measured to be 283 people.

### Participants

The participants were selected from the older adult population, presenting to health centers, via convenience sampling. The inclusion criteria were as follows: not taking anti-anxiety and anti-depression drugs, no diagnosis of cancer or other incurable diseases; no experience of death in first-degree relatives due to COVID-19; and no experience of a serious adverse event in the last six months. On the other hand, individuals who delivered incomplete questionnaires were excluded from the study.

### Data collections

In this study, data were collected using three questionnaires. The older adult demographic questionnaire included the participants’ age, sex, marital status, education level, and occupation. The second questionnaire was the Connor-Davidson Resilience Scale (CD-RISC). This questionnaire consists of 25 items, designed by Connor and Davidson in 2003. The scoring of this questionnaire is based on a Likert scale (completely false, 0; rarely true, 1; sometimes true, 2; often true, 3; and always true, 4). The minimum and maximum scores are zero and 100, respectively, and the cutoff point is 50. In other words, a score above 50 represents resilience in an individual, with higher scores (> 50) reflecting greater resilience, and vice versa. Connor and Davidson measured a Cronbach’s alpha coefficient of 0.89 for the CD-RISC. Also, the reliability coefficient, based on the test-retest method in a four-week interval, was estimated at 0.87 [37]. This scale has been validated in various studies [38, 39].

The third questionnaire was Templer’s Death Anxiety Scale (DAS). This scale, which was designed by Templer in 1970, consists of 15 items and five dimensions on the individual’s attitude toward death. It is scored from zero (“no death anxiety”) to 15 (“very high death anxiety”), with the midpoint set at 6–7 (cutoff point); a score higher than the cutoff point (7–15) represents high death anxiety, whereas a score lower than the cutoff point (0–6) denotes low death anxiety. For scoring, each true response is assigned one point, while each false response is assigned a score of zero; scoring is reversed for items 10, 11, 12, 13, 14, and 15 (true = 0, false = 1). The DAS scale has acceptable validity, and its Cronbach’s alpha coefficient is estimated at 0.83 [40]. Using the Kuder-Richardson formula, Templer reported the reliability of this scale to be 0.83 [41]. Besides, concurrent validity coefficients for its correlation with the manifest anxiety and depression scales were 0.27 and 0.40, respectively [42]. This scale has been validated in various studies [42–44].

### Data analysis

The collected data were analyzed in SPSS Version 22. Descriptive statistics, such as frequency, percentage, mean, and standard deviation (SD), were used to assess the demographic characteristics, as well as the questionnaire scores and dimensions. Also, t-test, analysis of variance (ANOVA), and Pearson’s correlation tests were used to calculate the mean differences and evaluate the correlation of variables. General linear model were used to quantify relationships and predicting among death anxiety, resilience, and demographic variables. A P-value less than 0.05 was considered statistically significant.

## Results

Of 283 older adult people participating in this study, 179 (63.3%) were male, and 104 (36.7%) were female, with a mean age of 67.29 ± 6.22 years. Also, 80.6% of the participants were married, 47.7% had elementary education, and 31.1% had a history of cigarette smoking. The older adult’s mean scores of resilience and death anxiety were 64.16 ± 9.60 and 6.33 ± 2.92, respectively.

According to Table 1, the mean resilience score was significantly associated with the older adult’s sex and employment status (P < 0.05). Male participants had higher score of resilience and lower score of death anxiety than female. The mean score of death anxiety was also significantly related to the older adult’s sex and employment status (P < 0.05). Participants with governmental job had higher score of resilience and unemployed participants had the highest score of death anxiety.

There was no statistically significant difference in term of smoking history, history of alcohol consumption and type of residence with the average score of death anxiety and resilience of the older adults (P < 0.05).

**Table 1:** The older adult’s mean (** ± ** SD) scores of resilience and death anxiety according to their demographic characteristics

				Resilience		Death anxiety	
Demographic characteristics		**n**	**%**	**Mean ± SD**	**Test result**	**Mean ± SD**	**Test result**
Sex	Male	179	63.3	65.73 ± 9.87	*P = 0.00	5.99 ± 2.93	*P = 0.010
	Female	104	36.7	61.45 ± 8.48		6.93 ± 2.90	
Marital status	Single	11	3.9	60.90 ± 7.63	**P = 0.387	5.18 ± 2.96	**P = 0.053
	Married	228	80.6	64.62 ± 9.88		3.28 ± 3.03	
	Widowed	43	15.2	62.60 ± 8.39		6.74 ± 2.23	
	Divorced	1	0.4	61.00		13.00	
Education	Elementary school	133	47.0	62.47 ± 9.84	**P = 0.071	6.55 ± 3.12	**P = 0.819
	Illiterate	2	0.7	75.00 ± 12.72		5.00 ± 0.00	
	Secondary school	39	13.8	64.7 ± 8.48		6.02 ± 2.66	
	High school diploma	56	19.8	65.58 ± 9.81		6.25 ± 3.04	
	Associate degree	29	10.2	66.10 ± 10.01		6.41 ± 2.98	
	Bachelor degree	24	8.5	65.95 ± 7.42		5.87 ± 2.30	
Employment status	Governmental job	108	38.2	66.129 ± 9.73	**P = 0.00	5.96 ± 2.80	**P = 0.004
	Self-employed	84	29.7	64.90 ± 9.84		5.91 ± 3.06	
	Unemployed	3	1.1	48.66 ± 11.50		9.66 ± 2.51	
	Housewife	88	31.1	61.56 ± 8.14		7.09 ± 2.86	
Smoking history	Yes	88	31.1	65.45 ± 9.90	*P = 0.12	6.07 ± 3.02	*P = 0.32
	No	195	68.9	63.57 ± 8.14		6.45 ± 2.91	
Alcohol consumption	Yes	3	1.1	71.00 ± 16.00	*P = 0.21	6.66 ± 2.30	*P = 0.84
	No	280	98.9	64.08 ± 9.52		6.33 ± 2.96	
Type of residence	Private house	267	94.3	64.19 ± 9.51	*P = 0.83	6.35 ± 2.94	*P = 0.70
	Child’s home	16	5.7	63.68 ± 11.18		6.06 ± 3.15	

*T-test, **ANOVA

There was a significant inverse correlation between the older adult’s mean scores of resilience and death anxiety (r=-0.290, P < 0.01); in other words, with an increase in resilience, the older adult’s death anxiety decreased.

In the analysis of the general linear model, the effect of demographic variables on resilience was investigated and the variables that were significant were included in the model, along with death anxiety. After entering these variables, death anxiety and the unemployed job became significant (Table 2).

**Table 2:** General linear models showing predictor of resilience and death anxiety based on correlated variables

Variable			*B	Standard Error	t	**Sig.	95% Confidence Interval	
							Lower Bound	Upper Bound
Death Anxiety			-0.796	0.187	-4.249	0.000	-1.165	-0.427
Sex	Male		13.815	11.083	1.247	0.214	-8.003	35.633
	Female***		-					
Job	Governmental job		0.255	2.899	0.088	0.930	-5.451	5.961
	Self-employed		0.971	5.332	0.182	0.856	-9.527	11.468
	Unemployed		15.456	6.498	-2.379	0.018	-28.247	-2.664
	Housewife***		-					

*Estimated value of unstandardized regression coefficient, **Significance, ***Reference

## Discussion

The results of the present study indicated the older adult’s low mean score of death anxiety. Similarly, a study by Khademi et al. revealed that the level of death anxiety was low in the older adult population [45]. Moreover, a study by Saina et al. in India reported that more than half of the older adult had moderate death anxiety, and a low percentage of them had severe death anxiety [46]; these results are consistent with the findings of the current study. As the prevalence of death anxiety was directly associated with the prevalence of COVID-19 in the investigated population, the lower level of death anxiety in the current research compared to the abovementioned studies can be attributed to the conditions of the present research, including vaccine accessibility, increased knowledge of COVID-19, and improved control and management of this disease in the general population.

In this regard, Rababa et al. found that the older adult’s mean score of death anxiety considerably increased during the COVID-19 pandemic [47]. Azgas et al. also reported that death anxiety was intensified during the COVID-19 pandemic, with the greatest impact observed in women, older adult, and medical staff [48]; however, these results are inconsistent with the current findings. One of the reasons for the discrepancy between the present and previous research can be differences in the study conditions, as well as the increased mortality rate of the older adult in the investigated countries.

Based on the present results, the older adult’s mean score of resilience was high. In this regard, Widzel et al. indicated that more than two-thirds of the older adult had moderate to high resilience, and a limited number of them showed low resilience [49]. In another study by Moradi et al., it was also found that the older adult’s level of resilience was high [50]; these results are consistent with the present study. On the other hand, in a study by Hajatnia et al., the older adult’s level of resilience was reported to be low [51], which is not in line with the present research. Since the mentioned study was conducted on older adult people living in nursing homes, the observed discrepancy between the current and previous research can be attributed to the presence of older adult people with lower resilience in nursing homes.

The results of the present study indicated a significant inverse correlation between resilience and death anxiety in the older adult; in other words, with an increase in the resilience score, the death anxiety score decreased. The results of a study by Paul et al. indicated a significant inverse correlation between death anxiety and resilience in individuals with COVID-19 [52]. Additionally, Haj-Hosseini et al. confirmed a significant inverse relationship between death anxiety and resilience [28], which is in line with the results of the present study. Generally, resilience culminates in the increased coping of an individual with unpleasant situations, as they accept and actively adapt to these circumstances; therefore, resilient individuals experience less psychological distress, such as tension, anxiety, and depression [30]. Conversely, the results of a study by Bahri et al. on patients with AIDS [53] contradict the current findings, which can be related to differences in the sample and design of the study.

In the present study, death anxiety was significantly different between male and female older adult, and the level of death anxiety was higher in women than men. Consistently, a study by Kavakli et al. indicated that death anxiety was higher in women than men during the COVID-19 pandemic [54]. Besides, a study by McLeod et al. showed that death anxiety was higher in females [55]. These results are in line with the present study, which showed that women are more aware of their emotions than men and express their fears more openly [56]. In contrast, Rababa et al. reported that the level of death anxiety was higher in males than females [47]. One of the reasons for this discrepancy can be the higher prevalence of COVID-19 and consequently, higher mortality rates among men over 60 years.

In the current study, the older adult with different occupations experienced different levels of death anxiety. Self-employed individual has high level of death anxiety in contrast with other occupations. Seyed Al-Shohadaee et al. reported similar results [30]. Having a steady job and a certain income creates economic security and as a result caused support for the mental health of the older adult [57]. Also, the findings of the present study indicated that the level of resilience was different between men and women, that is, resilience was higher in men. In this regard, Barkhordari Sharifabad et al. reported similar results [58]. On the contrary, Masoud et al. showed that women had higher resilience scores than men [59]; this difference can be related to different age groups of the investigated populations in the two studies. The current research suggested that the older adult with different occupations had different levels of resilience. Similar findings have been reported in the literature [30, 60]. Seemingly, financial independence and accessible welfare facilities lead to increased peace of mind and resilience. Therefore, the retired older adult people are less anxious, as they have permanent salaries, unlike self-employed older adult; consequently, they have a higher level of resilience [57].

Other important findings of this study include the positive predictive relationships of demographic status and the negative predictive relationship of death anxiety to resilience. These findings are consistent with the results of other studies and show as death anxiety increases, resilience decreases [61, 62].

Using of self-reported questionnaires, conducting of the study in a specific province of Iran, not having enough information about the current health status of the participants, lack of inclusion of all relevant covariates (such as type of medications, diagnosis, subjective health), and fairly small sample size can be considered as the possible limitations of this study.

## Conclusion

Overall, older adults had surprisingly high levels of resilience and low levels of death anxiety despite the COVID pandemic. Both death anxiety and resilience levels vary depending on sociodemographic parameters. As death anxiety and resilience seem to be inversely linked, resilience may be a promising protector against death anxiety that should be fostered in older adults.

## Data Availability

Data are available from the corresponding author on reasonable request.
